# The immediate effects of blood flow restriction training on upper limb muscle strength and fatigue level: a meta-analysis

**DOI:** 10.3389/fphys.2025.1521145

**Published:** 2025-07-01

**Authors:** Jian Wang, Jie Xu, Haiyang Liu, Lizhu Jiang

**Affiliations:** ^1^ Department of Physical Education, Ningbo University of Technology, Ningbo, China; ^2^ College of Education, Ningde Vocational and Technical College, Ningde, China

**Keywords:** blood flow restriction training, rate of perceived exertion, muscle strength, blood lactate, fatigue level

## Abstract

**Objective:**

Blood flow restriction training (BFR training) has gained recognition as a potentially effective intervention; however, its specific effects on upper limb strength and fatigue levels remain inadequately explored. This study aims to systematically assess the impact of BFR training on immediate upper limb strength and fatigue through a meta-analytic approach, with the goal of providing empirical evidence to inform its practical implementation in clinical and athletic settings.

**Methods:**

Search PubMed, CNKI, Web of Science and EBSCO databases, collect randomized controlled trials (RCTs) on the effects of BFR training on immediate upper limb strength and fatigue degree. Include and exclude literature through the Cochrane risk of bias tool. Employ Revman5.4 and Stata16.0 software for literature quality assessment and statistical analysis. Utilize sensitivity analysis and funnel plots to evaluate the stability of results and publication bias.

**Results:**

A total of 32 articles and 524 subjects were incorporated. Meta-analysis revealed that upper limb BFR training significantly increased immediate muscle strength [*SMD* = 0.36, 95%*CI* (0.02, 0.70), *P* = 0.04]. Moreover, BFR training had a significant impact on fatigue degree [*SMD* = 1.38, 95%*CI* (0.81, 1.94), *P* < 0.00001]. Given the high heterogeneity of the two groups of studies (*I* ^2^ = 77%, 88%), subgroup analysis demonstrated that BFR training could significantly enhance bench press strength [*SMD* = 0.40, 95%*CI* (0.07, 0.74), *P* = 0.02]. When the exercise intensity was 40%–70% of one repetition maximum (1RM) [*SMD* = 1.16, 95%*CI* (0.83, 1.50), *P* < 0.0001] and the compression intensity was ≥60% AOP [*SMD* = 0.64, 95%*CI* (0.24, 1.03), *P* = 0.0002], the effects on immediate upper limb strength reached the maximum and were statistically significant respectively. Subgroup analysis of fatigue degree indicated that BFR training could increase the blood lactate value [*SMD* = 2.15, 95%*CI* (1.06, 3.23), *P* < 0.0001] and subjective fatigue degree (rating of perceived exertion, RPE) [*SMD* = 1.11, 95%*CI* (0.36, 1.87), *P* = 0.004] of the subjects. Maximal effort [*SMD* = 2.61, 95%*CI* (2.14, 3.07), *P* < 0.0001] and compressive strength of ≥60% AOP [*SMD* = 2.64, 95%*CI* (1.35, 4.22), *P* = 0.001] had the greatest and significant effects on fatigue degree.

**Conclusion:**

Upper limb BFR training can significantly enhance bench press strength. BFR training with 40%–70% 1RM and ≥60% AOP is more likely to promote immediate upper limb strength. Compared with resistance training without compression, exhaustive training may have a negative effect on upper limb muscle strength. BFR training combined with maximal effort and ≥60% AOP can increase the blood lactate value and subjective fatigue degree of the subjects.

**Systematic Review Registration:**

http://inplasy.com, identifier: INPLASY202430008.

## 1 Introduction

Among numerous sports events, muscle strength is one of the important factors affecting athletic performance (Boullosa et al., 2013). Within athletic training regimens, athletes frequently implement tailored exercise protocols aligned with individualized performance objectives, which often necessitate the incorporation of high-intensity strength training to optimize outcomes. (Cleary Christopher and Cook Summer 2020). For people without strength training experience and those in rehabilitation, high-intensity strength training may increase the risk of training injuries (Minniti et al., 2020), but blood flow restriction (BFR) training provides an alternative method for such people.

BFR training also known as KAATSU training, is a training method in which a certain external pressure is applied to the proximal end of the limbs using a compression cuff or elastic bandage to reduce arterial blood inflow and block venous blood outflow, and is combined with aerobic exercise or resistance training to enhance muscle function (Marty et al., 2015). Compared with high-intensity strength training, the training effect of BFR training is more derived from metabolic stress (Wang, 2021). Studies have shown that the combination of BFR training and low-intensity exercise can produce beneficial muscle adaptations and has good effects in improving muscle mass and strength (Abe et al., 2009), promoting rehabilitation treatment and preventing disuse atrophy (Faltus et al., 2019; Miller et al., 2021).

Warm-up exercises have been widely studied. Scientific warm-up programs can effectively improve athletic performance (Wu et al., 2020). In recent years, in terms of immediate muscle strength improvement, most studies have confirmed that training combined with resistance and BFR can become an effective warm-up method for enhancing explosive power (Wang J. et al., 2023). In upper extremity BFR training, some studies have shown that BFR training can induce upper extremity muscle activation and PAP (Liu et al., 2024). However, BFR training is not without flaws. The use of compression devices may cause discomfort. Blood flow restriction pressure can accelerate the accumulation of muscle metabolites (Bielitzki et al., 2024). Subjects may feel fatigue more quickly during training (Schwiete et al., 2021). At the same time, blood lactate levels increase during BFR training, which easily exacerbates body fatigue (Christiansen et al., 2020). Therefore, external training methods and internal blood restriction degree may be important factors affecting BFR training. The immediate effects of BFR training on upper extremity strength and fatigue degree still need to be further confirmed.

At present, research on BFR training mainly focuses on lower limb muscles, and there are relatively few studies on upper limb muscles (Doma et al., 2020). In previous studies, BFR training mainly focused on the long-term adaptive effects on muscle strength and explosive power, while there is a relative lack of research on acute BFR resistance training (Mccann and Flanagan, 2010; Miller et al., 2018). At the same time, there is a lack of relatively unified standards for the intervention scheme of BFR training method combined with upper limb resistance training (Wang et al., 2022). Based on the above analysis, this study compares the changes in upper limb muscle strength, rating of perceived exertion (RPE), and blood lactate (BLA) of subjects after combining different degrees of blood restriction intensity with different types of resistance training, aiming to provide a scientific basis for optimizing upper limb training programs, improving training effects, and better understanding muscle fatigue caused by BFR training.

## 2 Materials and methods

### 2.1 Search strategy

The following databases were searched: PubMed, CNKI, Web of Science, and EBSCO. A total of 32 articles were retrieved, with the search period spanning from the establishment of each database to 3 July 2024. The English search terms employed were as follows: (“blood flow restriction training” or “KAATSU training” or “BFR” or “pressure training”) and (“muscle strength” or “explosive force” or “muscle recruitment” or “explosive power” or “fatigue” or “tiredness” or “weariness” or “upper limbs” or “upper extremities”), and (“RCT”).

### 2.2 Inclusion and exclusion criteria

#### 2.2.1 Inclusion criteria

Study Design: Published RCTs evaluating the effects of BFR training on upper limb muscle strength and fatigue. Participants: Healthy individuals (≤45 years), with no age restrictions. (no diagnosed musculoskeletal, cardiovascular, or metabolic conditions). Intervention: Experimental groups must involve BFR training applied to the upper limbs, with explicit documentation of BFR parameters (cuff pressure, occlusion intensity). Comparator: Control groups may include non-BFR training (traditional resistance training) or no training. Outcomes: Studies must report quantitative data for at least one muscle strength outcome (1RM) or one fatigue-related outcome (blood lactate, RPE). Accessibility: Full-text articles published in peer-reviewed journals.

#### 2.2.2 Exclusion criteria

Non-RCT Designs: Non-randomized trials, observational studies, reviews, or case reports. Irrelevant Interventions: Studies not involving BFR training or focusing on non-upper limb interventions. Population Exclusions: Studies involving patients with chronic diseases, or animal models. Outcome Exclusions: Studies lacking quantitative data on muscle strength or fatigue outcomes. Duplicate or Inaccessible Data: Duplicate publications or studies with incomplete/unavailable full texts.

### 2.3 Data extraction

**FIGURE 1:**
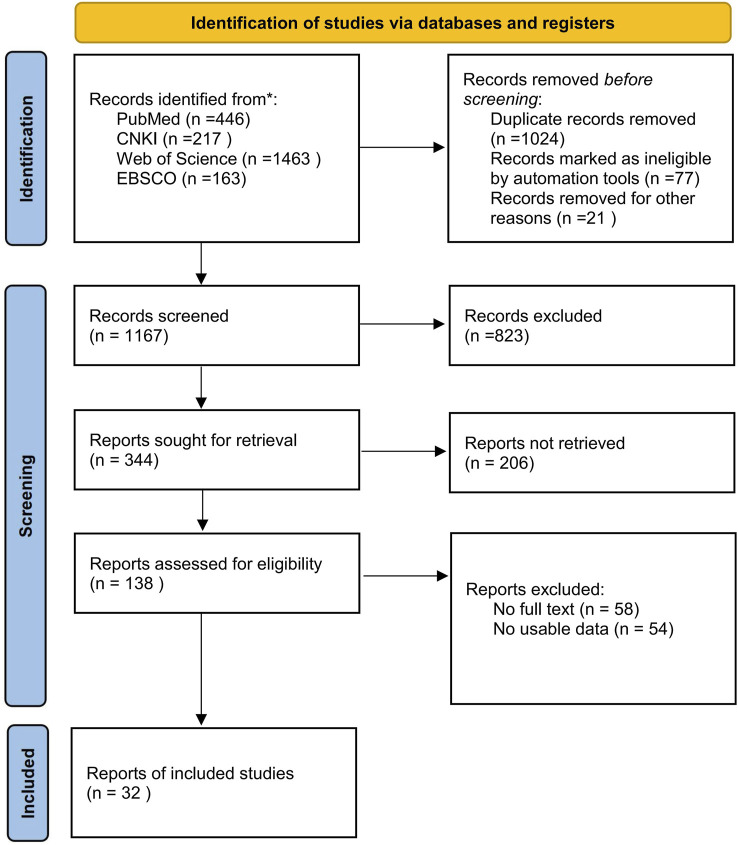
Flow diagram of literature selection.

The retrieved literature was uniformly imported into EndNote software and screened by two researchers, J.W. and J.X., separately. Discrepancies were resolved through discussion and negotiation or third-party judgment. The literature screening and inclusion steps are shown in Figure 1. Finally, 32 articles were included.

Two researchers extracted data from the literature that met the inclusion criteria using a customized form, mainly including the following contents.

1. General information: first author, publication year.
2. Sample characteristics: research subjects, age, sample sizes of the experimental group and the control group.
3. Experimental characteristics: intervention measures of the experimental group and the control group (including training methods, training intensity, and cuff intensity).
4. Extracted indicators: Quantitative indicators of upper limb muscle strength and fatigue degree.

### 2.4 Statistical analysis

Statistical analysis was performed using Revman 5.4. The standardized mean difference (*SMD*) and 95% confidence interval (95% *CI*) were selected as the effect size for the combined effect size. The quality of the included literature was evaluated using the Cochrane Risk of Bias Assessment Tool. Before the combined meta-analysis, a heterogeneity test was conducted first. The homogeneity test (*Q* test, test level a = 0.1) was used for the heterogeneity test. The value range of *I* ^2^ was between 0% and 100%. When *I* ^2^ > 50% and *p* < *a*, it indicated the presence of heterogeneity, and the random effects model was selected for the meta-analysis. Conversely, the fixed effects model was selected. The subgroup analysis was employed to address heterogeneity, and STATA 16.0 was utilized for sensitivity analysis to examine the stability of the results. The Egger test and funnel plot were adopted to verify the existence of publication bias.

## 3 Results

### 3.1 Study characteristics

A total of 32 publications were incorporated into this study. All of them were randomized controlled trials (RCTs), involving 524 subjects of mixed gender and with an age range of 16–45 years. The basic characteristics are presented in Table 1.

**TABLE 1:** Characteristic of studies included in systematic review and meta-analysis.

Study	Country	Age (years)	N (E.G.,/CG)	Intervention (E.G.,/CG)	Plan (BFR intensity)	Outcome extracted
Zhang (2023)	China	20.20 ± 0.92	10/10	BFR/No BFR	4 sets of 8 repetitions of bench press at 30% 1RM (140 mmHg)	BP↑ RPE↑
Serrano-Ramon et al. (2023)	Spain	23.6 ± 4.1	14/14	BFR/No BFR	3 repetitions of bench press at 60% 1RM (80% AOP)	BP↑
Duarte de Oliveira et al. (2023)	Brazil	27.1 ± 5.0	16/16	BFR/No BFR	4 sets of 30-15-15-15 times 20% 1RM bench press (150 mmHg)	RPE↑
Lei (2023)	China	23.67 ± 1.73	10/10	BFR/No BFR	3 sets of 8 repetitions of bench press at 70% 1RM (180 mmHg)	RPE↑
Ahmadi et al. (2021)	Canada	24.7 ± 4.9	13/13	BFR/No BFR	30-s maximal voluntary contraction of elbow flexion(100% AOP)	MVC ↓ VAS↑
Borges et al. (2018)	Portugal	22.0 ± 2.0	62/62	BFR/No BFR	4 groups of 30-15-15-15 times 20% 1RM elbow flexors (137.90 ± 11.8 mmHg)	MVC↑
Zhao (2023)	China	19 ± 1.23	20/20	BFR/No BFR	15-20 repetitions of pull-ups (150 mmHg)	MVC BB↑ RPE↑
Neto et al. (2017)	Brazil	18 ± 0.82	10/10	BFR/No BFR	4 sets of 30-15-15-15 times 20% 1RM bench press (163.80 ± 10.52 mmHg)	RPE NS BP NS BLA↑
Jessee et al. (2017a)	USA	18∼35	29/29	BFR/No BFR	4 groups of 30-15-15-15 times 30% 1RM elbow flexors (30% AOP)	MVC↓
Jessee et al. (2017b)	USA	24 ± 2	14/14	BFR/No BFR	4 groups of 30-15-15-15 times 30% 1RM elbow flexors (40% AOP)	RPE↑
Che et al. (2022)	China	23.6 ± 3.1	10/10	BFR/No BFR	4 groups of 30-15-15-15 times 30% 1RM bench press (160 mmHg)	RPE↑
Madarame et al. (2010)	Japan	26.3 ± 3.1	9/9	BFR/No BFR	The first set of 30 repetitions was followed by the second and third sets to failure with 30% 1RM of elbow flexors (130 mmHg)	BLA↑
Mattocks et al. (2019)	USA	21.5 ± 2.4	20/20	BFR/No BFR	4 sets of 30-s 15% 1RM elbow flexors (80% AOP)	RPE↑
Zhiyuan et al. (2022)	China	28.7 ± 7.1	10/10	BFR/No BFR	4 sets of 30 repetitions of elbow flexors at 30% 1RM (30 mmHg)	BLA NS RPE↑
Roehl et al. (2023)	USA	29.4 ± 4.3	15/15	BFR/No BFR	3 repetition of common rotator cuff exercises at 1RM (170 mmHg)	VAS ↑
Henrique et al. (2019)	Brazil	23.0 ± 2.67	13/13	BFR/LL	4 sets of 8 repetitions of elbow flexors at 30% 1RM (20 mmHg)	EF1↑ RPE↑
Wang et al. (2023b)	China	30∼45	15/15	BFR/No BFR	4 sets of 15 repetitions of elbow flexors at 20∼30% 1RM (30 mmHg)	BLA↑
Buckner et al. (2018)	USA	22 ± 2	22/22	BFR/No BFR	4 sets of elbow flexors to failure at 15% 1RM (40% AOP)	MVC BB↓
Lambert et al. (2014)	USA	18∼45	16/16	BFR/No BFR	4 groups of 30-15-15-15 times 20% 1RM dumbbell scaption (50% AOP)	EF↑
AmiltonVieira et al. (2015)	USA	22.2 ± 3.8	13/13	BFR/No BFR	3 sets of elbow flexors to failure at 50% 1RM (110 mmHg)	RPE↑ BLA↑
Dankel et al. (2017)	USA	26 ± 3	10/10	BFR/No BFR	2 sets of elbow flexors to failure at 70% 1RM (70% AOP)	RPE↑
Yasuda et al. (2015)	Japan	27 ± 5	10/10	BFR/No BFR	4 sets of elbow flexors to failure at 20% 1RM (160 mmHg)	RPE↓ BLA↑
Carla Florianovicz et al. (2020)	Brazil	21 ± 1.67	58/58	BFR/No BFR	10 sets of 6 repetitions of wrist curl at 40% 1RM (140 ± 12.79 mmHg)	GS↑
Wilk et al. (2020)	Poland	23.2 ± 2.66	12/12	BFR/No BFR	1 repetition of bench press at 1RM (100% AOP, 135 ± 16 mmHg)	BP↑
Salagas et al. (2022)	Greece	25.8 ± 6	12/12	BFR/No BFR	4 sets of 12-s rapid bench press at 60% 1RM (100% AOP, 146 ± 15 mmHg)	RPE↓
Rodrigues et al. (2023)	Brazil	29.9 ± 5.9	15/15	BFR/No BFR	1 repetition of bench press at 1RM (170 mmHg)	BP↑ RPE↓
Sun et al. (2020)	China	25.2 ± 4.0	8/8	BFR/No BFR	6 sets of dumbbell curls to failure at 50% 1RM (200 mmHg)	EF↓
Thiebaud et al. (2014)	USA	22.4 ± 3.2	9/9	BFR/No BFR	4 groups of 30-15-15-15 times 30% 1RM elbow flexors (120 mmHg)	MVC BB↑ MS↑
Zhang (2021)	China	22.5 ± 2.7	20/20	BFR/No BFR	6 sets of 8 repetitions of elbow flexors at 30% 1RM (110 mmHg)	EF↑
Yasuda et al. (2009)	Japan	24.1 ± 3.2	10/10	BFR/No BFR	4 groups of 30-15-15-15 times 20% 1RM elbow flexors (160 mmHg)	RPE NS
Yasuda et al. (2014)	Japan	23∼41	9/9	BFR/No BFR	4 groups of 30-15-15-15 times 20% 1RM elbow flexors (170–260 mmHg)	RPE NS BLA↑
Zhiyuan et al. (2022)	China	19.7 ± 3.2	10/10	BFR/No BFR	4 groups of 30-15-15-15 times 30% 1RM bench press (160 mmHg)	BP↑

NS, no statistical significance; MVC, maximum voluntary contraction; ↑ represents a significant increase; ↓ represents a significant decrease; BP, maximum strength of bench press; RPE, rate of perceived exertion; MS, muscle soreness; BLA, blood lactate; VAS, visual analog scale; MS, muscle soreness, EF, maximum strength of elbow flexors, LL, low load exercise; BB, biceps brachii; GS, grip strength; AOP, arterial occlusion pressure.

### 3.2 Study quality assessment

**FIGURE 2:**
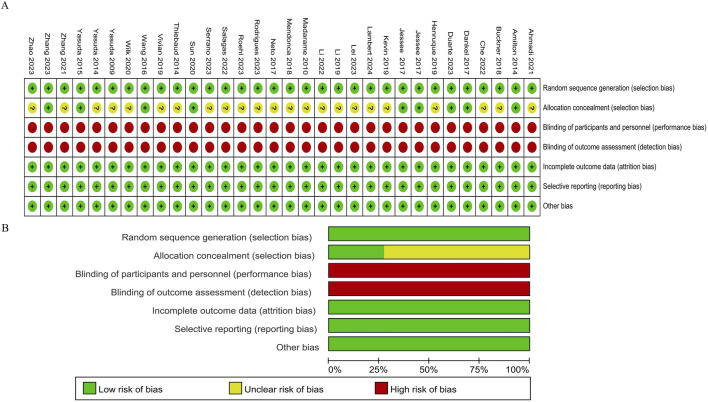
Methodological quality graph and summary of the included studies: **(A)** Risk of bias summary; **(B)** Risk of bias graph.

The quality of the literature was evaluated by referring to the Cochrane Risk of Bias Assessment Tool (Higgins and Altman, 2007). Seven aspects, including random sequence generation, allocation concealment, participant blinding, outcome blinding, incomplete outcome data, selective reporting, and other bias, were assessed using Review Manager 5.4 software (Figures 2A,B). Twenty-four articles failed to clearly describe whether the allocation personnel strictly adhered to random allocation, and 32 articles were at high risk of bias in blinding due to the signing of informed consent forms before the experiment.

### 3.3 Upper limb muscle strength

**FIGURE 3:**
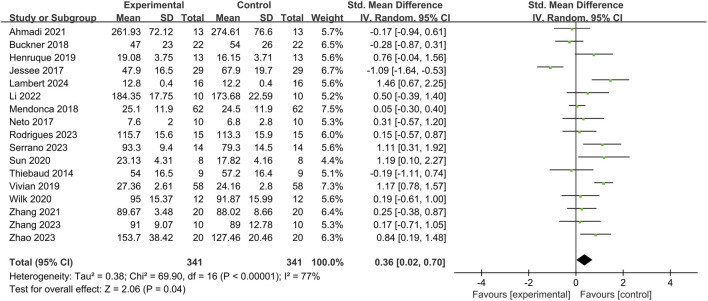
Effect of BFR training on muscle strength.

Among the 32 included articles, 17 articles compared the upper limb muscle strength of 341 subjects before and after BFR training (Figure 3). Heterogeneity test showed that *I* ^2^ = 77% > 50%, and *P* of *Q* test <0.01, which means there is strong heterogeneity among the literatures. Therefore, random effects were selected for meta-analysis. The pooled *SMD* value of 17 studies is 0.36, and the 95% confidence interval is from 0.02 to 0.70, and it is statistically significant (*Z* = 2.06, *P* = 0.04), indicating that BFR training can significantly enhance the muscle strength of the upper limbs.

### 3.4 Degree of fatigue

**FIGURE 4:**
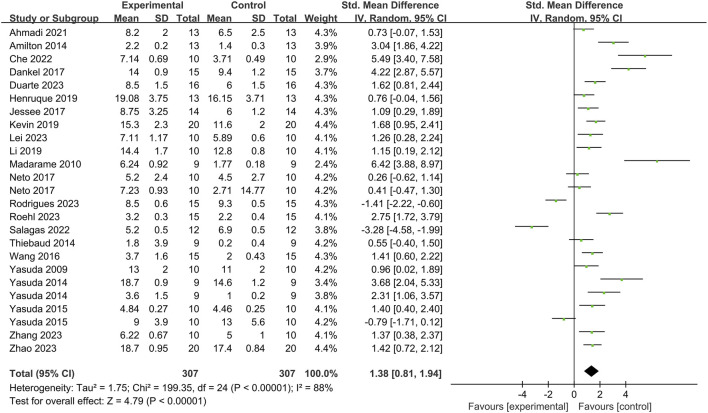
Effect of BFR training on fatigue.

Regarding the impact of BFR training on fatigue degree, a total of 25 articles and 307 subjects were included (Figure 4). The heterogeneity test result is *I* ^2^ = 88% > 50%, and *p* of *Q* test <0.01, indicating significant heterogeneity among studies. Therefore, a random effects model is used for analysis. The results show that the combined effect size of BFR training on fatigue degree is *SMD* = 1.38, 95%*CI* (0.81, 1.94), which is significant (*Z* = 4.79, *p* < 0.0001), suggesting that BFR training can affect fatigue degree to a large extent.

### 3.5 Subgroup analysis

Based on the data of the impact of BFR training on upper limb muscle strength, subgroup analyses were conducted on Exercise mode, Exercise intensity, and Compressive strength respectively according to the characteristics that may cause heterogeneity (Table 2).

**TABLE 2:** Subgroup analysis of BFR training on upper limb muscle strength.

Research features	Subgroup standard	Study (sample)	*SMD*	95%*CI*	*P*	*I2* (%)	*P* (Heterogeneity)
Exercise mode	Bench press	6 (71)	0.40	0.07, 0.74	0.02	0	0.54
	Elbow flexors	9 (192)	0.17	−0.31, 0.64	0.50	79	<0.0001
	Pull-up	1 (20)	0.84	0.19, 1.48	0.01	N	N
	Wrist curl	1 (58)	1.17	0.78, 1.57	<0.0001	N	N
Exercise intensity	≤30% 1RM	9 (168)	0.30	0.09, 0.52	<0.006	34	<0.15
	40%∼70% 1RM	4 (80)	1.16	0.83, 1.50	<0.0001	0	0.99
	Maximal effort	6 (73)	−0.54	−0.87, −0.21	0.002	49	0.11
	Self weight	1 (20)	0.84	0.19, 1.48	0.01	N	N
Compressive strength	≤40% AOP	3 (64)	−0.24	−1.22, 0.75	0.64	86	0.0009
	40%∼60% AOP	3 (45)	0.52	−0.40, 1.43	0.27	76	0.01
	≥60% AOP	11 (232)	0.50	0.16, 0.84	0.002	63	<0.004

N, no applicable.

**TABLE 3:** Subgroup analysis of BFR training’s impact on upper limb fatigue.

Research features	Subgroup standard	Study (sample)	*SMD*	95%*CI*	*P*	*I2* (%)	*P* (Heterogeneity)
Measurement tool	RPE	16 (204)	1.11	0.36, 1.87	0.004	90	<0.0001
	Muscle soreness	1 (9)	0.55	−0.40, 1.50	0.25	N	N
	VAS	2 (28)	6.88	−6.33, 20.10	0.31	98	<0.0001
	Blood lactate	6 (66)	2.15	1.06, 3.23	0.0001	82	<0.0001
Exercise intensity	≤30% 1RM	11 (131)	1.38	0.38, 2.38	0.007	91	<0.0001
	40%∼70% 1RM	4 (46)	2.85	2.24, 3.47	<0.0001	0	0.61
	Maximal effort	8 (95)	2.61	2.14, 3.07	<0.0001	0	0.51
	Self weight	2 (35)	−1.14	−1.75, −0.53	0.0002	0	0.32
Compressive strength	≤40% AOP	5 (67)	0.76	−0.01, 1.54	0.05	88	<0.0001
	40%∼60% AOP	7 (101)	1.66	0.96, 2.35	<0.0001	75	0.0005
	≥60% AOP	13 (139)	2.64	1.35, 4.22	0.0001	88	<0.0001

N, no applicable.

Subgroup analysis of different Exercise modes shows that the within-group heterogeneity of the group with Exercise mode as Elbow flexors has increased (*I* ^2^ = 79%), compared with the overall combined effect (*I* ^2^ = 82%), suggesting strong heterogeneity among the literatures of this Exercise mode. The number of studies on Pull-up and Wrist curl is small and lacks representativeness. Among them, Bench press has the highest homogeneity and is statistically significant (*I* ^2^ = 0%, *p* = 0.02 < 0.05), indicating that upper limb BFR training can significantly improve bench press strength.

Subgroup analysis of Exercise intensity shows that the heterogeneities of the three groups are 34%, 0%, and 49% respectively, which are all reduced compared with the overall combined effect (*I* ^2^ = 77%). It is speculated that different Exercise intensities may be one of the influencing factors leading to heterogeneity among studies. Compared with ≤30% 1RM and body weight exercises, the exercise intensity of 40%–70% 1RM reaches the maximum effect size *SMD* = 1.16 and is significant (*p* < 0.01), indicating that BFR training combined with 30%–70% 1RM can significantly enhance muscle strength. On the contrary, when the exercise intensity reaches Maximal effort, the effect size is negative *SMD* = −0.54 and is statistically significant *p* = 0.002 < 0.05, suggesting that exhaustive BFR training may temporarily have a negative effect on upper limb muscle strength.

The Compressive strength subgroup shows that the between-group heterogeneity of the group with applied BFR pressure ≤40% AOP has increased (*I* ^2^ = 86%), suggesting strong heterogeneity among the literatures under this applied BFR pressure. Among them, the combined effect size of the group with ≥60% AOP is the highest and is significant (*SMD* = 0.64, *P* = 0.002), indicating that BFR training with ≥60% AOP can significantly improve upper limb muscle strength.

For the research on fatigue degree, the author suspects that the sources of heterogeneity are the inconsistency of measurement tools, exercise intensity, and compressive strength. Therefore, subgroup analysis is carried out according to the source ofheterogeneity (Table 3).

According to different measurement tools, among the four groups of Rate of Perceived Exertion (RPE), muscle soreness, Visual Analogue Scale (VAS), and blood lactate, the heterogeneity between studies of the RPE and VAS scales is relatively high (90% and 98% respectively). Among them, the combined effect sizes of RPE (*SMD* = 1.11, *P* = 0.004) and blood lactate (*SMD* = 2.15, *P* < 0.0001) are statistically significant and positive, indicating that upper limb BFR training can significantly increase the RPE and blood lactate of the subjects.

In different Exercise intensity subgroups, the heterogeneities of the four groups are 91%, 0%, 0%, and 0% respectively. Compared with the overall combined effect (*I* ^2^ = 88%), the 40%–70% 1RM, Maximal effort, and Self weight groups show a completely homogeneous state. It is speculated that different exercise intensities may be an influencing factor leading to heterogeneity among studies. When the exercise intensity is Maximal effort, the effect size is the largest and significant (*SMD* = 2.61, *P* < 0.0001). When the exercise intensity is Self weight, the effect size is negative and statistically significant (*SMD* = −1.14, *p* = 0.0002). The results show that upper limb BFR training combined with maximal effort exercise intensity will significantly increase the fatigue degree of the subjects, while upper limb BFR training combined with body weight exercise intensity is not easy to cause fatigue in the body.

The Compressive strength subgroup shows that the heterogeneities of the three groups are 88%, 75%, and 88% respectively. Among them, when the applied BFR pressure is ≥ 60% AOP, the effect size is the highest and significant (*SMD* = 2.64, *P* = 0.0001). This indicates that under different applied BFR pressures, there are differences in the impact on the fatigue degree of the subjects. A higher applied BFR pressure (≥60% AOP) can significantly increase the fatigue degree of the subjects, while the effects under other applied BFR pressures are relatively weaker.

### 3.6 Sensitivity analysis

Sensitivity analysis was conducted on the included studies, and individual studies in each group were excluded to determine heterogeneity.

Table 4 shows that the combined effect size of BFR training on upper limb muscle strength is [*SMD* = 0.36, 95% *CI* (0.02, 0.70), p = 0.04, *I* ^2^ = 77%]. After excluding the studies of Jessee, Lambert, and Vivian, *I* ^2^ decreased to 65%, 76%, and 70% respectively, and heterogeneity was reduced to a certain extent. After excluding individual studies, the range of combined effect size *SMD* is between 0.29 and 0.46, and the p-values are all less than 0.1, which fully shows that no single literature will pose a threat to the results of meta-analysis.

**TABLE 4:** Combined effects of upper limb muscle strength after excluding individual studies.

Study	*SMD*	95%*CI*	*P*(Merge effect)	*I* ^*2*^(%)
Ahmadi et al. (2021)	0.39	0.04, 0.75	0.03	72
Buckner et al. (2018)	0.40	0.05, 0.76	0.03	77
Henrique et al. (2019)	0.34	−0.02, 0.69	0.07	78
Jessee et al. (2017a)	0.46	0.17, 0.75	0.02	65
Lambert et al. (2014)	0.29	−0.05, 0.63	0.09	76
Zhiyuan et al. (2022)	0.35	−0.01, 0.71	0.05	78
Borges et al. (2018)	0.39	0.01, 0.76	0.05	78
Neto et al. (2017)	0.36	0.00, 0.72	0.05	79
Rodrigues et al. (2023)	0.37	0.01, 0.74	0.04	78
Serrano-Ramon et al. (2023)	0.31	−0.04, 0.67	0.08	77
Sun et al. (2020)	0.32	−0.03, 0.67	0.07	78
Thiebaud et al. (2014)	0.39	0.03, 0.74	0.03	78
Carla Florianovicz et al. (2020)	0.29	−0.03, 0.61	0.08	70
Wilk et al. (2020)	0.37	0.01, 0.73	0.04	79
Zhang (2021)	0.37	0.00, 0.73	0.05	79
Zhang (2023)	0.37	0.01, 0.73	0.04	78
Zhao (2023)	0.33	−0.33, 0.69	0.07	78
Overall	0.36	0.02, 0.70	0.04	77

Table 5 indicates that after excluding individual studies on the impact of individual BFR on fatigue degree, the range of the standardized mean difference (*SMD*) is between 1.25 and 1.53, and the p-values are all less than 0.01. Compared with the combined effect size [*SMD* = 1.38, 95% *CI*(0.81, 1.94), *p* < 0.0001, *I* ^2^ = 88%], the research results remain unchanged, suggesting that this study has good stability.

**TABLE 5:** Fatigue merger effect after excluding individual studies.

Study	*SMD*	95%*CI*	*P*(Merge effect)	*I* ^*2*^(%)
Ahmadi et al. (2021)	1.41	0.82, 2.00	<0.0001	88
AmiltonVieira et al. (2015)	1.31	0.74, 1.87	<0.0001	88
Che et al. (2022)	1.25	0.70, 1.80	<0.0001	87
Dankel et al. (2017)	1.26	0.71, 1.81	<0.0001	87
Duarte de Oliveira et al. (2023)	1.37	0.78, 1.96	<0.0001	88
Henrique et al. (2019)	1.41	0.82, 2.00	<0.0001	88
Jessee et al. (2017b)	1.40	0.80, 1.99	<0.0001	88
Mattocks et al. (2019)	1.37	0.78, 1.96	<0.0001	88
Lei (2023)	1.39	0.80, 1.97	<0.0001	88
Zhiyuan et al. (2022)	1.39	0.80, 1.80	<0.0001	88
Madarame et al. (2010)	1.25	0.70, 1.79	<0.0001	87
Neto et al. (2017)	1.43	0.84, 2.01	<0.0001	88
Neto et al. (2017)	1.42	0.84, 2.01	<0.0001	88
Rodrigues et al. (2023)	1.48	0.95, 2.02	<0.0001	86
Roehl et al. (2023)	1.32	0.75, 1.89	<0.0001	88
Salagas et al. (2022)	1.53	1.01, 2.04	<0.0001	85
Thiebaud et al. (2014)	1.42	0.83, 2.00	<0.0001	88
Wang et al. (2023b)	1.38	0.79, 1.97	<0.0001	88
Yasuda et al. (2009)	1.40	0.81, 1.99	<0.0001	88
Yasuda et al. (2014)	1.29	0.73, 1.86	<0.0001	88
Yasuda et al. (2014)	1.34	0.76, 1.92	<0.0001	88
Yasuda et al. (2015)	1.38	0.79, 1.96	<0.0001	88
Yasuda et al. (2015)	1.47	0.90, 2.03	<0.0001	87
Zhang (2023)	1.38	0.79, 1.97	<0.0001	88
Zhao (2023)	1.38	0.79, 1.98	<0.0001	88
Overall	1.38	0.81, 1.94	<0.0001	88

The same literature name refers to different research results included in the same literature.

### 3.7 Publication bias

**FIGURE 5:**
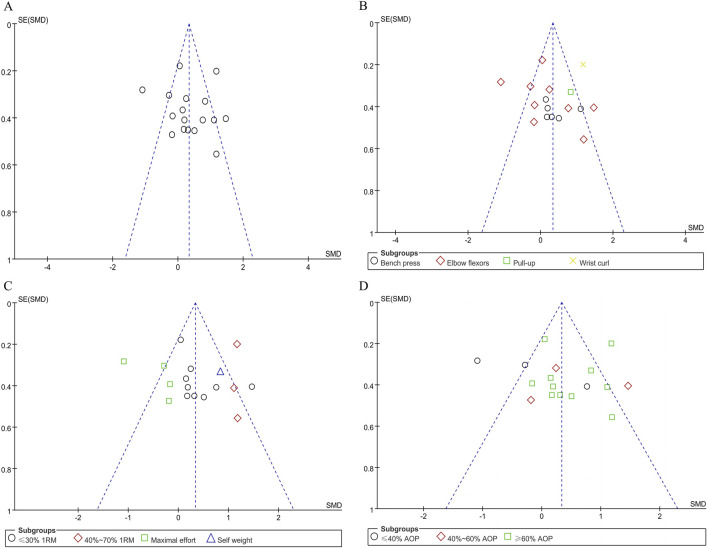
Funnel plot of upper limb muscle strength: **(A)** Combine funnel chart; **(B)** Exercise mode; **(C)** Exercise intensity; **(D)** applied BFR pressure.

**FIGURE 6:**
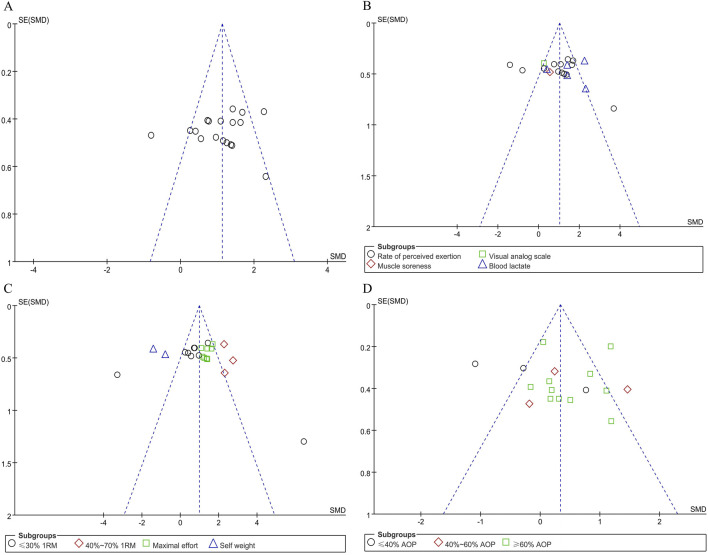
Funnel plot of upper limb fatigue level: **(A)** Combine funnel chart; **(B)** Measurement tool; **(C)** Exercise intensity; **(D)** applied BFR pressure.

Offset tests were conducted separately according to the subgroups of the effects of BFR training on upper limb muscle strength and fatigue degree. The publication bias of the included literatures was investigated by drawing funnel plots (Figures 5, 6). The test results show that the funnel plots are basically symmetrical, and the p-values in the Egger test are all greater than 0.05. This fully indicates that within the scope of this study, there is no obvious publication bias in the studies on the effects of BFR training on upper limb muscle strength and fatigue degree, and the research results have high reliability.

## 4 Discussion

### 4.1 Impact of BFR training on upper limb muscle strength

Strength is critical for athletic performance (Young, 2006). This study examined acute effects of BFR training combined with various protocols on upper limb strength. Meta-analysis of 18 studies demonstrated significant positive effects (*p* < 0.05), indicating BFR training’s efficacy in enhancing muscular strength. The mechanism involves limb occlusion-induced distal congestion, creating localized hypoxia and lactic acid accumulation that recruits fast-twitch fibers (Yazgan et al., 2024), consistent with previous findings (AmiltonVieira et al., 2015). Notably, high-threshold motor unit activation depends on contraction dynamics and intramuscular oxygen levels (Lau et al., 2023; Toshiaki et al., 2016). BFR-induced metabolite accumulation may accelerate neuromuscular activation through: 1) metabolic stimulation of group III/IV afferents (Jnsson et al., 2024), and 2) cross-bridge cycle modulation (Kubota et al., 2008), highlighting its potential for strength training.

Sensitivity analysis confirmed result stability, though substantial heterogeneity (*I* ^2^ = 82%) suggests methodological variations across studies. This variance may stem from multiple factors. Firstly, BFR training programs vary in applied pressure, intensity, duration, and frequency, all of which can impact training outcomes (Jennings et al., 2005). Secondly, different measurement methods, with their diverse levels of accuracy and reliability, also affect results (Batista Mauro et al., 2011). Thus, this study performed subgroup analysis based on potential sources of heterogeneity.

#### 4.1.1 Exercise mode

Subgroup analysis indicates that BFR combined with bench press enhances immediate upper limb strength more effectively than other exercises. This aligns with post-activation potentiation mechanisms, where controlled training (e.g., squats) induces neuromuscular excitation and rapid power gains (Batista Mauro et al., 2011), suggesting BFR’s potential to trigger similar effects.

Heterogeneity varies across muscle groups: elbow flexors show high variability due to individual differences and training protocol nuances, while limited studies on pull-ups and wrist curls hinder comprehensive assessment. Notably, bench press exhibits minimal heterogeneity (*I* ^2^ = 0%), likely attributable to its standardized protocols and easier variable control in study designs.

#### 4.1.2 Exercise intensity

For different exercise intensities, combining BFR training with 30%–70% of 1RM can significantly enhance upper limb muscle strength. While high-load exercises activate more muscle fibers (Hamada et al., 2000), moderate-intensity BFR training effectively promotes muscle growth and strength. However, at maximal effort, the effect size turns negative, suggesting that exhaustive BFR training may temporarily reduce upper limb strength, likely due to excessive fatigue impairing normal muscle function.

The subgroup analysis reveals complex yet insightful findings. The heterogeneity within the three subgroups is notably lower compared to the overall combined effect, suggesting that exercise intensity is likely a significant factor contributing to the variability observed across studies. Previous research supports this, showing that differing exercise intensities can variably impact muscle strength (Smirmaul et al., 2017), leading to divergent outcomes in study results.

#### 4.1.3 BFR pressure

As the applied BFR pressure changes, the impact of BFR training on immediate upper limb strength also varies. Compared with the compressive strength of 0%–60% AOP, BFR training with ≥60% AOP can significantly enhance immediate upper limb strength. Compared with low-intensity compressive strength, high-intensity blood flow restriction increases the metabolic pressure of muscles and promotes the release of growth factors, etc., thereby more effectively stimulating the growth of upper limb muscle strength (Laurentino et al., 2016).

Pressures below 50% AOP may inadequately accelerate muscular fatigue, a key driver of BFR adaptations (Rolnick, 2024). Low-intensity compression elicits a weaker muscle stress response, and heterogeneity increases in groups with ≤40% AOP. This variability may stem from individual differences, varied measurement methods, and subtle training program adjustments, potentially limiting BFR training’s impact on immediate upper limb strength.

### 4.2 Impact of BFR training on fatigue degree

Muscle fatigue, a physiological phenomenon during exercise, is characterized by reduced muscle strength, exercise capacity, and subjective tiredness (Constantin, 2024). Our findings confirm that BFR training can significantly influence the degree of fatigue. This result can be understood from multiple perspectives. During exercise, reduced oxygen and nutrient supply to muscles shifts energy production to anaerobic pathways (Kof and Strojnik, 2008). Local hypoxia and metabolite accumulation alter nerve cell ion channel function, impairing nerve impulse conduction (Gamboa and Andrade, 2012). Some studies have pointed out that in BFR training, the firing frequency of motor neurons is reduced and the activation degree of muscles is decreased (Martin et al., 2015; Fahs et al., 2015), which may be one of the reasons for fatigue.

Although BFR training significantly impacts fatigue, the results show high heterogeneity, likely due to varied intervention methods, training intensities, durations, and applied pressures. Differences in method reliability and effectiveness, as well as higher compression levels causing severe local hypoxia and metabolic stress, may exacerbate fatigue (Godawa et al., 2012). To address this, subgroup analyses were conducted based on measurement tools, exercise intensities, and BFR pressure.

#### 4.2.1 Measurement tool

Upper limb BFR training can significantly increase the RPE and blood lactate. For fatigue detection, different measurement tools often lead to differences in results. During BFR training, the insufficient energy supply to muscles and the increased accumulation of metabolites such as lactic acid will cause increased muscle fatigue, soreness, and weakness (Fatela et al., 2016). Studies have shown that BFR training can cause metabolic changes, and an increase in blood lactate levels is often associated with an increase in fatigue (Jessee et al., 2018). Therefore, BFR training can induce the degree of fatigue of the subjects to a certain extent.

However, different subjective fatigue assessment tools and methods are used in different studies, such as rate of perceived exertion, visual analog scale, etc. There may be differences in the reliability and effectiveness of these methods, and subjects have different subjective feelings of fatigue. This may account for the high heterogeneity of the RPE and VAS scales in this study. Additionally, blood lactate measurements, influenced by metabolic enzyme activity, cardiopulmonary function, and muscle fiber composition, can vary due to instrument and methodological differences (Coco et al., 2014). High heterogeneity likely arises from the combined impact of these factors.

#### 4.2.2 Exercise intensity

BFR training under different exercise intensities has different effects on the degree of fatigue. Among them, the 40%–70% 1RM, Maximal effort, and Self weight groups show a completely homogeneous state, which further supports the view that different exercise intensities may be one of the influencing factors leading to heterogeneity between studies (Smirmaul et al., 2017). When the exercise intensity is Maximal effort, the fatigue degree of the subjects is significantly increased (*SMD* = 2.61, *P* < 0.0001). High-intensity exercise often exerts greater metabolic pressure and neuromuscular load on the body. In exhaustive exercise, neurotransmitter depletion and slowed nerve conduction speed are more likely to lead to fatigue (Taylor and Romer, 2008).

The results indicate that upper limb BFR training combined with self-weight exercise intensity is less likely to induce fatigue (*SMD* = −1.14, *p* = 0.0002). Self-weight exercises, being low-intensity, result in slower energy consumption and rely primarily on aerobic metabolism, which may not trigger significant metabolic or neuromuscular fatigue responses (Hody et al., 2011). In summary, the varying effects of upper limb BFR training across exercise intensities stem from differences in metabolic stress and neuromuscular load. These factors interact, causing fatigue to rise significantly under maximal effort but remain minimal during self-weight exercises.

#### 4.2.3 Cuff pressure

The relationship between arterial occlusion pressure intensity and fatigue in BFR training is complex (Ilett et al., 2019). Higher AOP may induce greater fatigue due to enhanced blood flow restriction, leading to muscle hypoxia and metabolite accumulation (Rolnick et al., 2024). Progressive training under these conditions can lead to energy depletion and lactic acid accumulation, exacerbating muscle fatigue and soreness (Pincivero and Campy, 2004).

This relationship is moderated by several factors. First, individual variability significantly influences fatigue responses to cuff pressure (Santos et al., 2019), with some individuals demonstrating greater tolerance to higher pressures. Second, training parameters (duration, frequency) interact with AOP intensity to modulate fatigue levels. Finally, interventions such as warm-up routines, recovery protocols, and nutritional strategies can mitigate fatigue perception (Sucharit et al., 2019), thereby reducing the direct correlation between AOP intensity and fatigue.

### 4.3 Study limitations

Despite rigorous screening and exclusion, several limitations remain. Ethical guidelines require participants to provide informed consent, disclosing study details and intervention specifics, which inherently compromises blinding. In this review, 33 studies could not implement blinding due to this requirement. While this methodological limitation exists, it is unlikely to severely distort results, given the emphasis on participant safety and transparency. Additionally, although BFR training has been shown to improve strength and power, the lack of standardized protocols leads to variability in training programs and testing methods, contributing to data heterogeneity. Future research should refine experimental designs to better understand the impact of factors like training status and exercise intervals.

## 5 Conclusion

BFR training significantly enhances upper limb muscle strength while concurrently exacerbating fatigue levels during acute exercise sessions. BFR training combined with bench press is more effective in enhancing immediate upper limb muscle strength. BFR with 40%–70% 1RM and ≥60% AOP is more beneficial for upper limb immediate strength. Compared to resistance exercise without BFR training, exhaustive training might exert a negative influence on upper limb muscle strength. Maximal effort combined with ≥60% AOP BFR training can increase blood lactate and subjective fatigue.

## Data Availability

The original contributions presented in the study are included in the article/supplementary material, further inquiries can be directed to the corresponding author.
